# Multiorgan WU Polyomavirus Infection in Bone Marrow Transplant Recipient

**DOI:** 10.3201/eid2201.151384

**Published:** 2016-01

**Authors:** Erica A. Siebrasse, Nang L. Nguyen, Melisa J. Willby, Dean D. Erdman, Marilyn A. Menegus, David Wang

**Affiliations:** Washington University School of Medicine, St. Louis, Missouri, USA (E.A. Siebrasse, D. Wang);; University of Rochester Medical Center, Rochester, New York, USA (N.L. Nguyen, M.A. Menegus);; Centers for Disease Control and Prevention, Atlanta, Georgia, USA (M.J. Willby, D.D. Erdman)

**Keywords:** WU polyomavirus, immunosuppression, epithelial cells, tropism, viruses, transplant

## Abstract

WU polyomavirus (WUPyV) was detected in a bone marrow transplant recipient with severe acute respiratory distress syndrome who died in 2001. Crystalline lattices of polyomavirus-like particles were observed in the patient’s lung by electron microscopy. WUPyV was detected in the lung and other tissues by real-time quantitative PCR and identified in the lung and trachea by immunohistochemistry. A subset of WUPyV-positive cells in the lung had morphologic features of macrophages. Although the role of WUPyV as a human pathogen remains unclear, these results clearly demonstrate evidence for infection of respiratory tract tissues in this patient.

## Abstract 2

Virus was detected in the lung and trachea of a deceased patient.

Polyomaviruses are small, circular, double-stranded DNA viruses. Infection with BK polyomavirus (BKPyV) or JC polyomavirus causes substantial illness and death in immunocompromised populations, including transplant recipients and HIV patients (*1*). Infection with either virus is typically asymptomatic unless the host is immunocompromised. Since 2007, a total of 11 additional human polyomaviruses have been discovered. Two of these viruses (Merkel cell carcinoma polyomavirus and trichodysplasia spinulosa–associated polyomavirus) have also been implicated as human pathogens in the context of immunosuppression (*2*,*3*); the former causes Merkel cell carcinoma, a rare but aggressive skin cancer (*3*), and the latter is associated with trichodysplasia spinulosa, a rare skin disease seen in transplant recipients (*2*). Several of the other new human polyomaviruses (e.g., human polyomavirus 9 and New Jersey polyomavirus) were also initially identified in immunocompromised patients (*4*,*5*).

In 2007, WU polyomavirus (WUPyV) was discovered in a child in Australia with pneumonia (*6*). Although the virus has yet to be implicated in human disease, epidemiologic studies have shown that 69%–80% of persons (*7*–*9*) are seropositive for this virus; infection probably occurs during early childhood. In addition, viral DNA has been detected in blood, feces, respiratory tract secretions, tonsils, and cerebrospinal fluid (*10*). Although these studies have contributed to a better understanding of WUPyV, only 1 has explored the in vivo tropism of the virus or described the detection of viral antigen in tissue (*11*). That study determined that WUPyV was present in epithelial cells from a bronchoalveolar lavage from a lung transplant recipient with Job syndrome. A complete understanding of the types of cells and tissues in which the virus replicates is critical for identifying potential diseases with which it may be associated. However, the propensity for viruses of the family *Polyomaviridae* to cause disease only within the context of immunosuppression makes disease association particularly challenging. We describe molecular characterization and immunohistologic and microscopic localization of WUPyV in tissues from a deceased patient who had had viral pneumonitis.

## Materials and Methods

### The Case

In January 2001, a 27-month-old girl was admitted to an upstate New York area hospital for a 5/6 human leukocyte antigen–matched cord blood transplant from an unrelated donor. The patient had been born by normal vaginal delivery after 40 weeks of gestation. Her medical history included leukocytosis at 3 months and splenomegaly at 6 months of age. Refractory juvenile myelomonocytic leukemia was diagnosed when she was 16 months of age, and she underwent splenectomy in September 2000. She had multiple infections before 2 years of age, including otitis media, a central vein catheter infection, and a urinary tract infection. She also demonstrated failure to thrive, developmental delay, mild pulmonic stenosis, and gastroesophageal reflux.

Three weeks after the bone marrow transplant, the child experienced fever; diarrhea; hepatomegaly; and erythema on her face, palms, and soles. She was evaluated for graft versus host disease, viral exanthema, and drug eruption. The results of skin biopsies performed at 4 weeks after transplantation ruled out graft versus host disease and drug eruption. A rectosigmoid biopsy performed at the same time as the skin biopsies showed mild stromal edema but was negative for adenovirus and cytomegalovirus by immunohistochemistry (IHC) staining. Throughout the course of the patient’s hospitalization, adenovirus was intermittently isolated from her feces and urine and influenza B virus was detected in her nose and throat. PCR testing of the blood for cytomegalovirus was consistently negative. Treatment included cyclosporine; Solu-medrol (Pharmacia & Upjohn LLC, New York, NY, USA); intravenous immunoglobulin; ribavirin; Zosyn (Pfizer Inc., New York, NY, USA); Flagyl (Pfizer Inc.); Flutamine (Schering-Plough, Kenilworth, NJ, USA); Tamiflu (Roche Pharmaceuticals, Nutley, NJ, USA); Demerol (Sanofi-Aventis U.S. LLC, Bridgewater, NJ, USA); Zofran (GlaxoSmithKline, Philadelphia, PA, USA); Phenergan (Wyeth, Madison, NJ, USA); Tylenol (Johnson & Johnson, New Brunswick, NJ, USA); albuterol; isradipine; Spironolactone (Mylan Pharmaceuticals, Morgantown, WV, USA); hemotransfusion; and platelet transfusion. Despite this aggressive therapy, the patient’s condition continued to deteriorate. On March 1, 2001, the patient was transferred to the pediatric intensive care unit because of respiratory failure. Chest radiographs revealed pulmonary edema. Her condition was stabilized 2 days later, but severe acute respiratory distress syndrome and distended abdomen developed on April 1. Treatment was continued and mechanical ventilation was added. A radiograph taken on April 15 (≈11 weeks after transplantation) revealed free air in the abdominal cavity, but the source was not identified. The patient died later that day; the probable cause of death was viral pneumonitis. An autopsy was performed.

### Electron Microscopy

Lung tissues were fixed in formalin and then postfixed in 2.5% glutaraldehyde/0.1 mol/L Millonig phosphate buffer before processing into epoxy resin for sectioning and film photography. Microscopic examination was performed with a Hitachi 7100 transmission electron microscope (Hitachi High-Technologies Science America Inc., Northridge, CA, USA). 

### IHC 

IHC was performed as described previously (*11*). In brief, formalin-fixed paraffin-embedded blocks of tissue were deparaffinized, rehydrated, and treated with 3% hydrogen peroxide. Antigen was retrieved, and samples were blocked in 1.5% normal horse serum. A mouse monoclonal antibody against viral protein (VP) 1 from WUPyV (WU-VP1) (NN-Ab06) or an isotype-matched control antibody (mouse IgG2b; BD Biosciences, San Jose, CA, USA; no. 557351) was incubated overnight. After incubation with the biotinylated antimouse IgG secondary antibody (Vector BA-2000; Vector Laboratories, Inc., Burlingame, CA, USA), slides were developed by using the Vectastain ABC kit (Vector Laboratories, Inc.; no. PK-6100) and DAB (Vector Laboratories, Inc.; no. SK-4100), counterstained with hematoxylin, dehydrated, cleared, and mounted.

### Double IHC 

The double IHC (dIHC) staining protocol was similar to the regular IHC protocol with the addition of several steps. After the blocking step, slides were incubated with NN-Ab06 and then the secondary antibody. Development was accomplished by using the ABC kit and ImmPACT SG (Vector Laboratories, Inc.; no. SK-4705). Tissues were blocked with avidin and biotin (Vector Laboratories, Inc.; no. SP-2001) then with 1.5% normal horse serum. Slides were incubated in the second primary antibody against CD68 (Dako, Glostrup, Denmark; no. M081401) and then in biotinylated antimouse IgG secondary antibody. The second set of staining was developed by using the ABC kit and 3,3′-diaminobenzidine, followed by dehydration. For the MUC5AC dIHC assay, staining was first performed with the monoclonal antibody against MUC5AC (Thermo Fischer, Rockford, IL, USA; no. MA1–38223), which was developed with 3,3′-diaminobenzidine. Staining with NN-Ab06 and development with SG substrate (Vector Laboratories, Inc.) followed the blocking steps. Tissues stained by using the dIHC protocol were not counterstained. Control staining with an IgG2b isotype antibody (for NN-Ab06) and an isotype antibody against IgG1 (for the CD68 and MUC5AC antibodies) was also performed.

### Nucleic Acid Extraction and PCR

DNA was extracted from formalin-fixed paraffin-embedded samples by using the QIAGEN BioRobot M48 workstation and MagAttract DNA Mini Kit (QIAGEN, Valencia, CA, USA). A quantitative real-time PCR (qPCR) assay for detection and viral load estimation of WUPyV/KIPyV was developed and used to screen the extracted DNA. Primer and probe sequences for the qPCR assay were forward 5′-GTAGCTGGAGGAGCAGAGGC-3′; 5′-CACCAAGRGCAGCTAARCCTTC-3′; and probe 5′-CTGGWTCTGGAGCTGCMATAGCWACTGGT-3′. qPCRs were performed by using iQ Supermix reagents (Bio-Rad, Hercules, CA, USA); each 25-µL reaction mixture contained 0.6 μmol/L forward primer, 0.3 μmol/L reverse primer, 0.1 μmol/L probe, and 5 μL nucleic acid extract. Amplification was conducted on an iCycler iQ Real-time Detection System (Bio-Rad). Thermocycling conditions consisted of 3 min at 95°C for activation of the iTaq DNA polymerase and 45 cycles of 15 s at 95°C and 1 min at 60°C. Extracts were also tested for human bocavirus (HBoV) by using a previously described qPCR assay (*12*). For distinguishing between WUPyV and KIPyV, conventional PCR and sequencing was performed by using primers forward 5′-GGAGCTGTAYAGAATGGAAAC-3′ and reverse 5′-TTCATCCAAYAGTGGAATTG-3′.

### Complete Genome Sequencing

Multiple segments of the WUPyV genome were amplified and sequenced by using overlapping primer sets (Table 1) designed from reference strain WU/Wuerzburg/02/07 (GenBank accession no. EU711057.1). Amplicons were directly sequenced by using the ABI Prism BigDye Terminator Cycle Sequencing Ready Reaction Kit, version 3.1, on an ABI 3130 XL DNA Sequencer (both from Applied Biosystems, Carlsbad, CA, USA). The complete genome sequence for isolate Rochester-7029 is available through GenBank under accession no. FJ794068.

**Table 1:** Primers used to sequence WU polyomavirus in lung tissue from a child with acute respiratory illness

Oligonucleotide	Sequence, 5′ → 3′ (reference, if applicable)
WUPYV-F1	GTAGCTGGAGGAGCAGAGGC
WUPYV-R1	CACCAAGAGCAGCTAAACCTTC
WUPYV-F2	CCACGCCCCCTACCCAG
WUPYV-R2	AATATGATGTCCAGATTCCATAGGC
WUPYV-F3	CCAAGGAGGTGGACTTAATATCCA
WUPYV-R3	ACCTGCCAGTGCCATTCC
WUPYV-F4	CGTTGGATATAAAGGGTCACCA
WUPYV-R4	GCCTCTGCTCCTCCAGCTA
WU seq F1	AGCTAAGCATGATTGACAGTGTG
WU seq R1	CAGACTCAACGGAGATGTCACA
WU seq F2	TCACTGTTATGTGCAGGAATGT
WU seq R2	ACAGCAAGCAATATGCCCATC
WU seq F3	TATTGGTGCTACCGTCTCGAAC
WU seq R3	GTGGATGGACTGGATATTAAGTC
WU seq F4	ATATATACAGCTTTAGCAGCAGATC
WU seq R4	CTTACTTGTTCAACTATAGCATTTACTG
WU seq F5	CAGTAGTTAATAGAGCAGTTAGTGAAGA
WU seq R5	TAGAAATGTCACTGTTTAGCTCTTC
WU seq F6	GATGGCTTTAATGCACTTAGTGATG
WU seq R6	GTAGCACACAGTAGTATCAGCATCAG
WU seq F7	ATTAGTAGCCCACTTAAAACTGCTG
WU seq R7	TCTGCCACCCATGATTCAATG
WU seq F8	GTTTATTCCAGTTCTGAAACACC
WU seq R8	GCAAATGAGACAAATTACTGGTTG
WU seq F9	CTTTATAAGCAGGTGTTTAATAAGC
WU seq R9	TAAAAGAAAGTCTGGATAAAACTCC
WU seq F10	TTCTTTCCAATACACAACTTTAGC
WU seq R10	GGTAAAACAACTGTTGCTGCT
WU seq F11	CTCCTACTTGACCTTTTACATCTTC
WU seq R11	CAACTCATAATAGACTTCATATGGAAC
WU seq F12	TCTTCTAGCTAATAAATCTTCTCTGG
WU seq R12	GTAATACATACCACCAAAGAAAAGG
WU seq F14	CAGCACTAACTCTATGTCTAAAAGG
WU seq R14	GGTGCTATAGAGAGTGGTTTGG
WU seq F15	CTCATTACATCTTAGTTCTTCTTCC
WU seq R15	AAGAATTTCATCCTGACAAAGG
WU seq F16	TCTACCTGTGAAGAGCTCCACAC
WU seq R16	CACATTCCTGCACATAACAGTG
WU seq F17	CTAAGCATGATTGACAGTGTGG
WU seq R17	TGATAGTGCCTCTGCTCCTC
AG0058	GCTCCACCTTGTGGCTGCTA (*6*)
AG0036	GCATTTACTGGGTCAGATTCC (*6*)
AG0035	TGCATTCTACCTGTGAAGAGC (*6*)
WU seq F6.5	GTACCACTGTCAGAAGAAACAGAG
WU seq F7.5	GATGTGCTAGGACTTGCTCC
WU seq R9.5	CCTCCAGGTATTGTAACAATGAATG
WU-C-4824-F	GGCACGGCGCCAACT (*13*)
WU-C-4898-R	CCTGTTGTAGGCCTTACTTACCTGTA (*13*)
WU 4422R	GAAATGCCTAAATCTCCTGGAG
WU 4341F	GTGTTGCCTGTGAACATTGTG
WU 4810R	AGACTGGGACATATGCTTAAAGG
WU 4571F	GCTTACCTGGTTAAGCCAAC
WU 4945R	GTGAAGTAGAAGAAGAAGTAAATCA
WU 5225R	AAAGCCTCAACTTTCTGAACTA
WU 783R	AAGCTCAGGTACTTTTGTTAGTACAG
PyVseq 844F	GGAGCTGTAYAGAATGGAAAC
PyVseq 2419R	TTCATCCAAYAGTGGAATTG

## Results

The most remarkable gross autopsy findings were bilateral pulmonary consolidation, acute tracheobronchitis, hepatomegaly with cholestasis, deep mucosal ulcerations throughout the small bowel, and prominent generalized lymphadenopathy. The most notable pathologic finding was heavy hemorrhagic foci in the lungs. Microscopic examination of the lungs confirmed the presence of interstitial emphysema with profound hemorrhage in the right upper, right middle, left upper, and left lower lobes. Multiple smudge cells and cells with Cowdry type A nuclear inclusions were identified inside reactive bronchial epithelium from both lungs. Similar inclusions were seen in epithelium of the trachea, bile duct, renal tubules, and urinary bladder. Small bowel mucosa revealed multifocal ulcerations and scattered inclusion-bearing cells in the epithelium. Findings after immunoperoxidase staining were negative for cytomegalovirus, adenovirus, influenza virus, human papillomavirus, respiratory syncytial virus, and simian virus 40, and in situ hybridization was negative for Epstein-Barr virus. Attempts to culture virus from the lung, liver, gastrointestinal tract, and lymph node tissues were unsuccessful. Gram-stained sections of lung, liver, and lymph node showed >25 neutrophils per low-power field without presence of organisms. Coagulase-negative *Staphylococcus* spp. grew from a blood culture (<30 CFU/mL) and culture of the gastrointestinal tract. No strictly anaerobic growth was observed.

**Figure 1:**
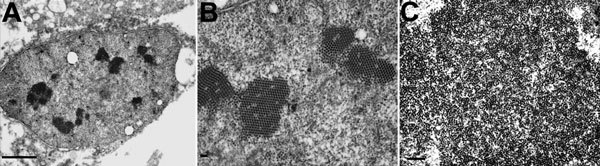
Electron micrographs of polyomavirus-like particles in lung from a child with fatal acute respiratory illness. A) Low-power view of a nucleus displaying multiple electron dense crystalline arrays. Scale bar indicates 0.5 μm; original magnification ×10,000. B) Higher-power magnification of nucleus in panel A. Scale bar indicates 100 nm; original magnification ×30,000. C) Large cluster of putative polyomavirus virions. Scale bar indicates 250 nm; original magnification ×20,000.

Electron micrographs of the lungs showed viral particles in the nuclei, many in para-crystalline arrays (Figure 1). The particles were 30.4–34.7 nm (mean 32.1 nm) in diameter. The diameter was less than the conventional diameter for polyomaviruses (45 nm), but the size of viral particles can vary according to the method of fixation and embedding (*14*). In addition, previously published electron microscopy findings for BKPyV particles indicated measurements of 30–50 nm (*15*). Despite the presence of viral particles indicative of polyomavirus, IHC on lung tissue with a primary antibody against simian virus 40, which is known to cross-react with BK and JC polyomaviruses, was negative. IHC for adenovirus and respiratory syncytial virus was also negative. Although electron microscopy indicated a probable viral infection in the patient’s lungs, because of the negative IHC results, no further testing was performed at that time.

### WUPyV in the Lungs

The recent discoveries of HBoV, another small, circular DNA virus, in 2005 and 2 new polyomaviruses, WU and KI (KIPyV), in 2007, in the respiratory tracts of children with acute respiratory illness prompted us to investigate the involvement of these viruses in this case. DNA extracted from formalin-fixed paraffin-embedded lung, liver, kidney, and gastrointestinal tissues was tested by qPCR for WUPyV, KIPyV, and HBoV. All 4 tissues were positive for WUPyV, whereas KIPyV and HBoV were not detected in any of the tissues. Virus loads estimated by qPCR were substantially higher in samples from the lung (cycle threshold 16.6) than in samples from the liver, kidney, and gastrointestinal tract (cycle threshold 30.2–30.8; Table 2). The entire WUPyV genome (designated Rochester-7029, GenBank accession no. FJ794068) was subsequently sequenced from lung tissue to 4× coverage (each base sequenced independently 4 times) by using multiple primer sets and found to be 5,306 bp. Compared with 79 complete WUPyV genome sequences available in GenBank, nucleotide identity scores for Rochester-7029 ranged from 0.970 to 0.985. Compared with the reference sequence, Rochester-7029 had 8 single-nucleotide polymorphisms, 5 of which were in coding regions. Two of these 5 were synonymous mutations. We predicted an amino acid change in VP2 and VP3 from glutamic acid to glutamine at positions 250 and 107, respectively. We also predicted amino acid changes in large T-antigen: glutamine to glutamic acid at position 134 and isoleucine to leucine at position 594. Of note, the Rochester-7029 genome contained a 77-bp terminal duplication in the large T-antigen as compared with the reference WUPyV genome, which was not predicted to have any effect on the size or sequence of the translated protein because it was located 3′ to the T-antigen stop codon.

**Table 2:** Summary of virus findings in tissue samples from child with acute respiratory illness*

Study	Lung	Liver	Kidney	Gastrointestinal
Intranuclear inclusions	Positive	Positive	Positive	Positive
Electron microscopy	Positive	Not performed	Not performed	Not performed	
WUPyV/KIPyV qPCR	Positive (16.6)	Positive (30.8)	Positive (30.4)	Positive (30.2)	
WUPyV PCR and sequencing	Positive	Positive	Positive	Positive	
KIPyV PCR and sequencing	Negative	Negative	Negative	Negative	
WUPyV IHC	Positive	Negative	Negative	Negative	
HBoV qPCR	Negative	Negative	Negative	Negative

*Numbers in parentheses indicate cycle thresholds from qPCR. HBoV, human bocavirus; IHC, immunohistochemistry; KIPyV, KI polyomavirus; qPCR, real-time quantitative PCR; WUPyV, WU polyomavirus.

**Figure 2:**
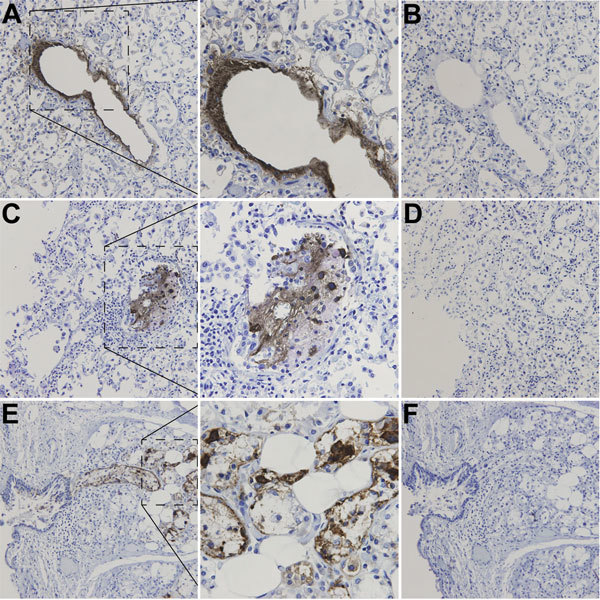
Immunohistochemical detection of WU polyomavirus viral protein 1 in respiratory tract of a child with fatal acute respiratory illness. Human lung tissue at original magnification of ×200, stained with a monoclonal antibody against WU polyomavirus viral protein 1 (designated NN-Ab06) (A, C) or an isotype control antibody (B, D). Human tracheal tissue at original magnification of ×200, stained with NN-Ab06 (E) or an isotype control antibody (F). The middle panels show insets from panels A, C, and E (dotted boxes) at higher original magnifications (×600).

After detection of WUPyV in the patient’s tissues by real-time qPCR, WUPyV-specific IHC with a previously described assay (*11*) was performed on available tissues (lung, liver, kidney, and gastrointestinal tract) to determine whether WU-VP1 antigen was also present (Figure 2). Liver, kidney, and gastrointestinal tissues were all negative (Table 2). Staining was observed in the lung (Figure 2, panels A, C) and the trachea (Figure 2, panel E), but no staining was observed in serial sections stained with an isotype control antibody (Figure 2, panels B, D, F). Serial sections stained with no primary or secondary antibodies were also negative (not shown). Overall, we saw 3 patterns of staining in the lung. In some cells, WU-VP1 staining was primarily in the nucleus. In others, the perimeter of the nucleus was strongly positive. And in others, the staining was diffuse, making it difficult to discern its position within cells. Of note, the tracheal staining was within a submucosal gland, where WUPyV tropism has not been previously described.

### WU-VP1 in CD68-Positive Cells

**Figure 3:**
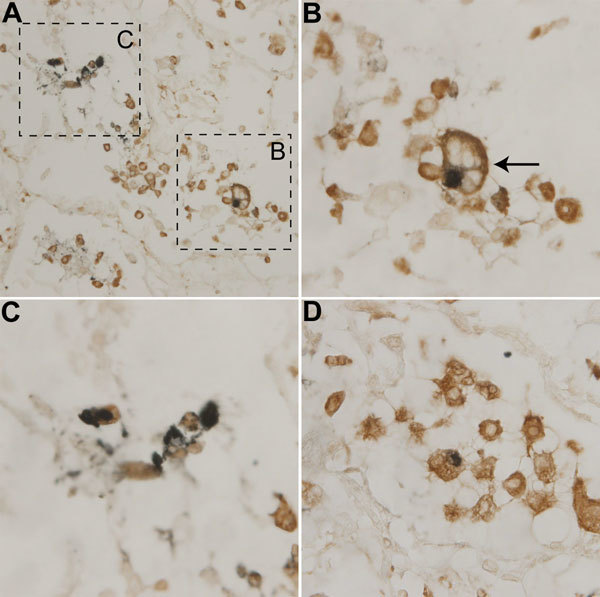
Detection of WU polyomavirus viral protein 1 in CD68-positive cells from a child with fatal acute respiratory illness. Lung tissue stained with NN-Ab06 (blue) and a monoclonal antibody against CD68 (brown). A) Tissue at original magnification of ×400. B) Closer view of cell from panel A consistent with a foamy macrophage (arrow). Original magnification ×1,000. C) Closer view of cells from panel A. Original magnification ×1,000. D) Different field of the tissue section with another double-positive cell. Original magnification ×1,000.

A recent article described detection of WU-VP1 in epithelial cells obtained from a bronchoalveolar lavage of a lung transplant recipient with Job syndrome (*11*). We anticipated that some WUPyV-positive cells in lung tissues of the patient reported here were also epithelial cells, but we did not explicitly confirm this suspicion because of the limited amount of lung tissue available. Instead we chose to explore additional hypotheses regarding potential tropisms of WUPyV because of our recent detection of KIPyV in CD68-positive cells (*16*). CD68 is a glycoprotein present on monocytes and macrophages. We performed dIHC testing by using the monoclonal antibody against WU-VP1 (NN-Ab06) and a monoclonal antibody against CD68, which primarily labels macrophages and monocytes. Cells positive for both WU-VP1 and CD68 were detected within the patient’s lung tissue (Figure 3). In addition, the cell shown in Figure 3, panel B (arrow) is morphologically consistent with a foamy macrophage, a specific morphotype of macrophage that is laden with lipid droplets in the cytoplasm (*17*). A serial section stained with isotype-matched antibodies (IgG2b for NN-Ab06 and IgG1 for the anti-CD68 antibody) was negative (not shown). In a recent study, KIPyV was also detected in a foamy macrophage (*16*). 

### WU-VP1 Antigen in Association with Mucin-Producing Cells

**Figure 4:**
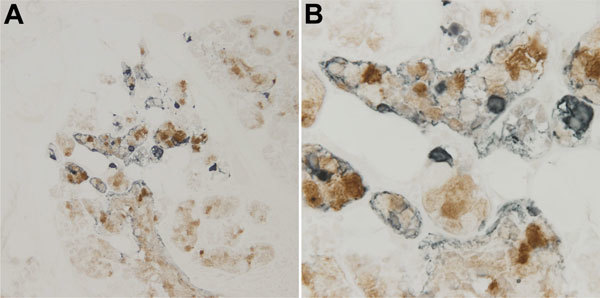
Detection of WU polyomavirus viral protein 1 in close proximity to MUC5AC-positive cells in the trachea of a child with fatal acute respiratory illness. Tracheal tissue stained with NN-Ab06 (blue) and a monoclonal antibody against MUC5AC (brown). A) Tissue at original magnification of ×200. B) Tissue at original magnification of ×600.

The initial IHC staining of tracheal tissue revealed positive cells within a submucosal gland. MUC5AC is the principal mucin produced by goblet cells, and MUC5B is produced by submucosal glands (*18*). We developed 2 dIHC assays: NN-Ab06 and a monoclonal antibody against MUC5AC and NN-Ab06 and a polyclonal antibody against MUC5B. Each assay was performed on control cell pellets (not shown). The MUC5AC dIHC assay yielded clearer staining, so we chose to apply this assay to the tracheal tissue from the patient. We detected WU-VP1–positive cells in association with a cluster of cells showing MUC5-AC positivity (Figure 4). It is unclear whether the 2 antigens colocalize to the same cell. WU-VP1–positive cells were also seen separate from MUC5AC-positive cells, suggesting that a subset of virus-positive cells do not produce mucin. We found 2 such areas in the tracheal tissue.

## Discussion

We describe a case of viral pneumonitis in a bone marrow transplant recipient who died in 2001. Before her death, influenza and adenovirus were identified from the patient. Although samples from the patient were initially tested for several viruses by IHC and culture, no agent was identified. Subsequent work since 2007 showed that multiple tissues from this patient were positive for WUPyV by real-time PCR and IHC, but the same tissues were negative for KIPyV and HBoV by PCR. To date, WUPyV has been the only virus detected in tissue samples from this patient. Crystalline lattices of polyomavirus-like particles were seen in the lung, which substantiated the high virus titers measured by qPCR. Collectively, these observations suggest a potential pathogenic role for WUPyV infection in this case. However, we cannot rule out the possibility that WUPyV infection was simply opportunistic in this severely immunocompromised patient.

Analysis of samples from this patient provided novel insights into fundamental properties of WUPyV infection in vivo. In the lungs, we detected WUPyV antigen in CD68-positive cells (probably of the macrophage/monocyte lineage) by immunohistochemistry. WUPyV is most closely related to KIPyV, and the viruses share many similarities, including an apparent tropism for CD68-positive cells (*16*). Other polyomaviruses have also been detected in cells of the monocytic lineage (*16*). We do not believe that this detection represents phagocytosis of other WUPyV-infected cell types because WU-VP1, a late-expressed protein, was detected in the nucleus of CD68-positive cells, suggesting an infection in this patient. Granted, we did not prove that infectious particles were produced.

WU-VP1 was also detected in close proximity to MUC5AC-positive cells in tracheal tissue. The detection of WUPyV in tracheal tissue was unexpected and expands the known tissue tropism of the virus. As previously mentioned, MUC5AC is a mucin primarily produced by goblet cells in the airway. Several viruses in glandular cells in the trachea have been described: adenovirus has grown in primary cultured peribronchial submucosal gland cells (*19*); rhinovirus has grown in human respiratory submucosal gland cells (*20*); severe acute respiratory syndrome coronavirus antigen and RNA have been detected in tracheal/bronchial serous gland epithelium (*21*); and BKPyV has been shown to replicate in salivary gland cells (*22**,**23*).

In conclusion, WUPyV was detected by multiple methods in the lung of a bone marrow transplant recipient who had viral pneumonitis at the time of death. Tracheal tissue from this patient was also positive for WU-VP1. Viral antigen was specifically detected in CD68-positive cells and in close association with MUC5AC-positive cells within a tracheal submucosal gland. The role of WUPyV as a human pathogen remains unclear, although the evidence for an infection of the respiratory tract in this patient is strong. This study expands our understanding of WUPyV biology and tropism beyond detection of virus in body fluids.
